# RetroRules: a database of reaction rules for engineering biology

**DOI:** 10.1093/nar/gky940

**Published:** 2018-10-13

**Authors:** Thomas Duigou, Melchior du Lac, Pablo Carbonell, Jean-Loup Faulon

**Affiliations:** 1 Micalis Institute, INRA, AgroParisTech, Université Paris-Saclay, 78350 Jouy-en-Josas, France; 2 SYNBIOCHEM Centre, Manchester Institute of Biotechnology, University of Manchester, Manchester M1 7DN, UK; 3 CNRS-UMR8030/Laboratoire iSSB, Université Paris-Saclay, Évry 91000, France

## Abstract

RetroRules is a database of reaction rules for metabolic engineering (https://retrorules.org). Reaction rules are generic descriptions of chemical reactions that can be used in retrosynthesis workflows in order to enumerate all possible biosynthetic routes connecting a target molecule to its precursors. The use of such rules is becoming increasingly important in the context of synthetic biology applied to *de novo* pathway discovery and in systems biology to discover underground metabolism due to enzyme promiscuity. Here, we provide for the first time a complete set containing >400 000 stereochemistry-aware reaction rules extracted from public databases and expressed in the community-standard SMARTS (SMIRKS) format, augmented by a rule representation at different levels of specificity (the atomic environment around the reaction center). Such numerous representations of reactions expand natural chemical diversity by predicting *de novo* reactions of promiscuous enzymes.

## INTRODUCTION

Engineering biology is a key technology at the forefront of the new industrial bio-economy (1). Bio-based production of chemicals and materials are alternatives to chemical processes both economically viable and ecologically sustainable. Promising new opportunities are now unveiled such as producing polymers using salt-tolerant *Halomonas* (2), spider silk fermentation with recombinant *Escherichia coli* or yeast to provide a non-fossil fuel biodegradable alternative to nylon (3). Progress in metabolic engineering and increasingly multiplexed high-throughput capabilities are harnessing synthetic biology to develop faster, more predictable, novel routes for fine and specialty chemicals production to deliver new chemical diversity towards scale-up and industrial manufacturing. The development of pathway design tools for metabolic engineering such as RetroPath2.0 (4), a retrosynthesis framework with the ability of predicting *de novo* reactions by replacing native enzymatic reactions through generic reaction rules, are expanding our ability to access natural chemical diversity. The variable representation of reaction rules mimics the natural capabilities of enzymes to promiscuously accept multiple substrates, allowing fine tuning of the pathway. Such a tool has been integrated with Selenzyme (5) a free online enzyme selection tool for metabolic pathway design that focuses on reaction rules. Therefore, there is a renewed interest in developing a comprehensive catalogue of biosynthetic capabilities based on reaction rules for the production of next generation bio-based chemicals. Recent efforts have focused on standardization of the technology through automated extraction of the reaction rules (6) and the use of machine learning to assist in the retrosynthetic search (7).

Current resources for reaction rules present some drawbacks, some focus on specific applications or have restricted access. For instance BNICE.ch (8), which is only available under a proprietary license; Chematica, that consists of reaction rules extracted from Reaxys for organic synthesis available under commercial license; reaction rules from MetRxn database (9) calculated through code available on GitHub (database of rules non available); MINE, a database that uses the BNICE reaction rules in order to generate new chemical structures for metabolomics data analysis, (10) but the reaction rules are not provided; and Transform-MinER, a reaction search tool based on KEGG reaction rules not available as a database (11). Moreover, another limitation is that traditional reaction rule-based systems do not take stereochemistry into account (12).

Here, we present RetroRules (https://retrorules.org), an open-source database of stereochemistry-aware reaction rules for engineering biology that integrates multiple data sources and automates the generation of ranked rules with variable specificity to reactants based on atomic environments around the reaction center.

## DATABASE IMPLEMENTATION AND CONTENT

### Data sources

Current release of RetroRules is based on MNXref version 3.0 (14). Sequence annotations were obtained by cross-referencing MNXref v3.0 with Rhea release 81 (15) and UniProt release 2017_04 (16).

### Database content

RetroRules provides a complete set of reaction rules spanning >16 000 biochemical transformations expressed at different levels of promiscuity.

The number of distinct reaction rules depending of diameter considered is summarized in Table 1. Even at the most permissive level (diameter = 2), the number of generated unique reaction rules exceed the number of biochemical reactions. This is due to the generation process in use considering that (i) reaction rules are generated for both directions of every reaction—which doubles the number of rules—and (ii) when a reaction involves multiple reactants that are not cofactors, a separate rule is generated for each substrate (see Materials and Methods section).

**Table 1.** RetroRules database content regarding the number of distinct reaction rules available at each diameter and depending whether or not stereochemistry is expressed in the reaction rules. The ‘All reaction rules’ column is the number of unique reaction rules from the two datasets

Diameter	Number of distinct reaction rules		
	Dataset not expressing stereochemistry	Dataset expressing stereochemistry	All reaction rules
2	18 607	23 391	39 016
4	24 241	28 906	49 933
6	27 686	31 734	56 544
8	29 276	33 128	59 770
10	30 158	33 944	61 627
12	30 762	34 519	62 854
14	31 219	34 932	63 786
16	31 559	35 258	64 453

Interestingly, while expressing stereochemistry in reaction rules opens up a wider range of possible transformations, such increase in reaction rules mainly occurs on EC classes that are typically involved in stereochemistry changes such as EC 5.1 and EC 5.2 (racemases and epimerases and *cis-trans*-isomerases, respectively), for which the number of distinct rules increases from 75 to 1171.

**Figure 1.**
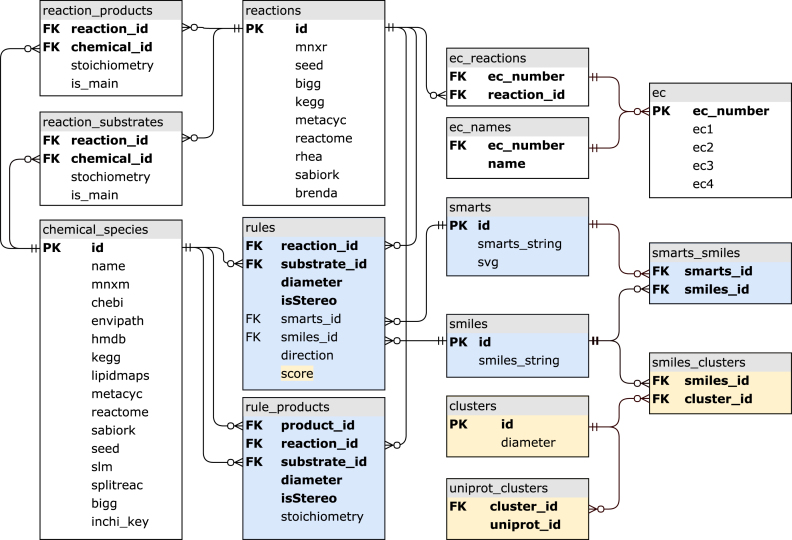
SQL schema used by RetroRules. The tables in white are the parsed meta-information from various datasets and their cross-references to others databases. The reactions and chemical_species tables are linked together through the bridge tables reaction_products and reaction_substrates. In blue are the tables associated with reaction rules. The rules table uniquely identifies a given rule with single reaction_id and substrate_id at a given diameter. The isStereo parameter determines if the rule describes the stereochemistry of the reaction or not. Each rule is associated with a single SMARTS and SMILES description of that reaction, and to link the two, a smarts_smiles table assures that one is able to easily find the corresponding SMILES description of a SMARTS and vice-versa. The rule_products table describes the products of a given rule. Lastly in yellow are the tables that were used to generate the score (also in yellow in the rules table) from the rules table (see the Reaction rule scoring section).

RetroRules is freely available to download as an SQLite database containing 15 tables and around 6 million entries that interrelate reactions, rules, metabolites, sequences, and a rule score based on biochemical diversity (Figure 1). At the center of the schema is the rules table that contains the information to uniquely describe a reaction rule from a mono-component reaction. A rule is uniquely associated with a given substrate, from a given reaction at a given diameter and, to determine if a given rule expresses stereochemistry of a reaction, the Boolean-type column isStereo. Because a reaction rule can have multiple products, the rule_products table includes all the unique identifiers of a rule (i.e. reaction_id, substrate_id, diameter and isSetereo) combined with the columns product_id and stoichiometry that describe a chemical species product for a rule and the number of occurrences of that product in the rule, respectively. The rule_products table thus contains a one-to-many relationship with the rules table. Since a given SMARTS or SMILES reaction rule description can apply to multiple reactions, substrates and diameters, both can be found in the smarts and smiles tables respectively. The remaining tables contain meta-information extracted from source databases. All protein sequences, chemical structures and parent reactions data are available in their respective public databases.

### Web server

The RetroRules database and related web services runs on our infrastructure. It is written in the Python 3 language using the Flask framework. The database content can be accessed through the website (Figure 3, https://retrorules.org), or through a REST API service (https://retrorules.org/api/v0.7/). In both cases the user may make ranked searches based on EC numbers, reaction IDs from metabolic databases (MetaNetX, Brenda, BiGG, KEGG, MetaCyc, Reactome, Rhea, SABIO-RK and The SEED) according to cross-references provided by MNXref version 3.0 (14), substrate names, or chemical structures in InChI format. Results of searches are returned as a sortable table that can be browsed online, exported into JSON or CSV, or directly accessed using the REST API. Providing a REST API facilitates the access to the RetroRules on-the-fly service or to the database using analytic pipelines and other bioinformatics tools.

The underlying chemical transformation with its associated atom-atom mapping for each individual rule can be inspected in detail through the RetroRules web service and a drawing of the reaction rule may be exported as SVG. The single reaction rule display gathers additional information not included in the query result table such as the sets of related mono-component reactions, substrate and reactions through MetaNetX identifiers, and the associated sequences through UniProt identifiers.

Lastly, in the circumstance that the user does not find the desired reaction rule in the precomputed database, RetroRules provides the user with the ability to generate custom rules using the *Do It Yourself* web service (https://retrorules.org/diy).

### Workflow integration

One of the advantages of the REST service is that it allows the use of RetroRules in a large variety of web-based applications. Complex queries involving RetroRules can be performed by employing a workflow framework such as KNIME (17) that allows the interconnection of several web services through well-defined steps, providing reproducibility both for protocols of experimental data analysis and *in silico* screening.

Notably, the RetroRules database can be queried in order to generate a set of reaction rules to be run through the RetroPath2.0 workflow for retrosynthesis (4). A REST query can be easily performed and its response converted into the standard format used for RetroPath2.0 including the reaction rule score, which is required for pathway ranking. A KNIME workflow providing such facility is available at https://www.myexperiment.org/workflows/5086. In the provided example, RetroRules was queried for reactions in the MetaCyc database. Resulting rules were then successfully used in RetroPath2.0 in order to identify heterologous biosynthetic routes for resveratrol in *E. coli*.

In yet another application example, RetroRules was integrated with the Selenzyme tool for enzyme sequence selection (5) so that for a given reaction, reaction rules are calculated at different diameters and top candidate sequences are retrieved for each rule. Such application can be considered in the scenario of some target reaction with no annotated sequences. RetroRules allows dissecting the reaction into its associated reaction rules of increasing generalizability by lowering the diameter. The rules are submitted to Selenzyme, which generates at each diameter the sets of most plausible candidate sequences according to the preselected ranking criteria. Even if the target reaction had no known annotation, a query for one of the reaction rules at some low diameter might deliver sequence candidates of target reaction activity because of enzyme promiscuity. An example of such type of nested queries for those reactions in the database having caffeine as reactant has been implemented using KNIME and is available at https://www.myexperiment.org/workflows/5082.

## MATERIALS AND METHODS

### Reaction rules calculation

**Figure 2.**
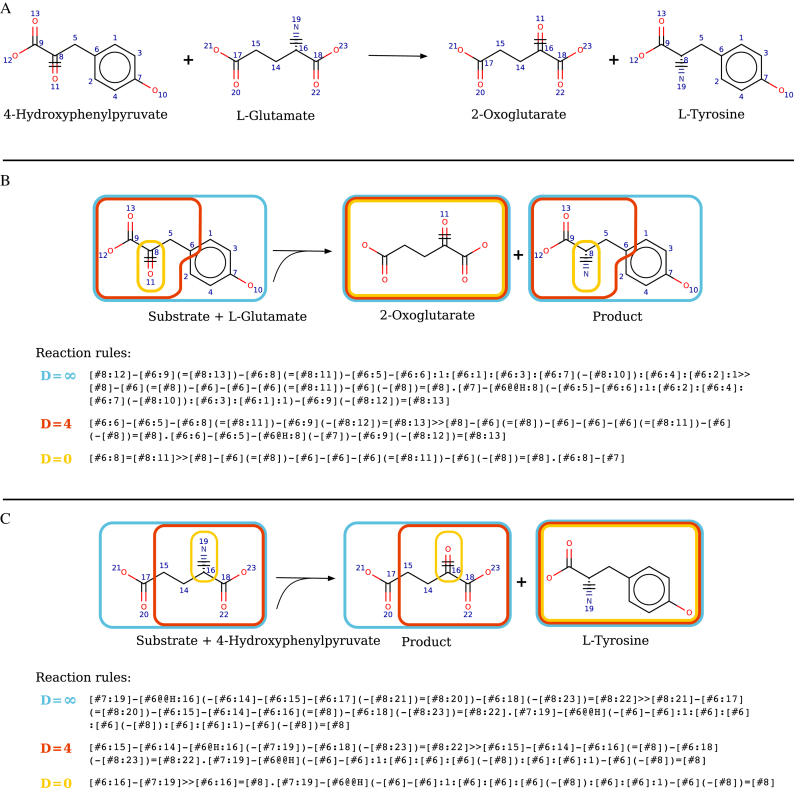
Calculation of reaction rules for EC 2.6.1.5. (**A**) The atom mapping highlights reacting atoms labelled 8, 11, 16 and 19. (**B**) Calculation of the EC 2.6.1.5 rules for diameters *D* = ∞, 4 and 0 modeling promiscuity on substrate 4-hydroxyphenylpyruvate. (**C**) Calculation of the EC 2.6.1.5 rules for diameters *D* = ∞, 4 and 0 modelling promiscuity on substrate l-glutamate.

Reaction rules were generated using the cheminformatics RDKit library http://www.rdkit.org/ in Python. In-depth description and validation of the generation process were described in (4). The procedure is outlined below:

1. Extract reaction information from metabolic databases. Filter out reactions that miss any structure from amongst involved compounds.
2. Remove reactions that do not trigger the modification of substrate (e.g. passive transport), are not balanced, or that involve compounds not fully characterized (e.g. *R*-groups).
3. Identify the reaction center (i.e. subpart(s) of substrate(s) that are transformed) based on an atom-atom mapping between substrates atoms and product atoms (AAM). Figure 2 shows reaction 2.6.1.5 with atom mapping, reacting atoms are those labelled 8, 11, 16 and 19.
4. Decompose multi-substrate reactions into mono-substrate components. There are as many components as there are substrates and each component gives the transformation between one substrate and the products. Each product must contain at least one atom from the substrate according to the AAM. This strategy enforces that only one substrate can differ at a time from the substrates of the reference reaction when applying the rule. Reaction decomposition to mono-substrate component are considered for both direction of reactions, leading during step 6 to the generation of reaction rules for both directions for every reaction and enabling utilization of reversed rules for retrosynthesis application. Panels B and C of Figure 2 show two mono-substrates component generated from reaction 2.6.1.5.
5. Optionally, substrate compounds that are cofactors (such as water, CO_2_, ATP, NADP, ions, …) can be ignored until the end of the procedure under the assumptions that such metabolites are available in the cell and that there is no gain to consider promiscuity on them. RetroRules' current release does perform a cofactor removal (Supplementary Table S1).
6. Compute the reaction rules using the reaction SMARTS formalism for each mono-substrate component. Perform rule SMARTS generation considering different diameters around the reaction center by removing from the components atoms that were not in the spheres around the reacting atoms. Panel B of Figure 2 shows reaction rules generated when considering three different diameters around the reacting atoms of l-glutamate (‘Substrate’). RetroRules' current release provides reaction rules for diameter 2 to 16.

**Figure 3.**
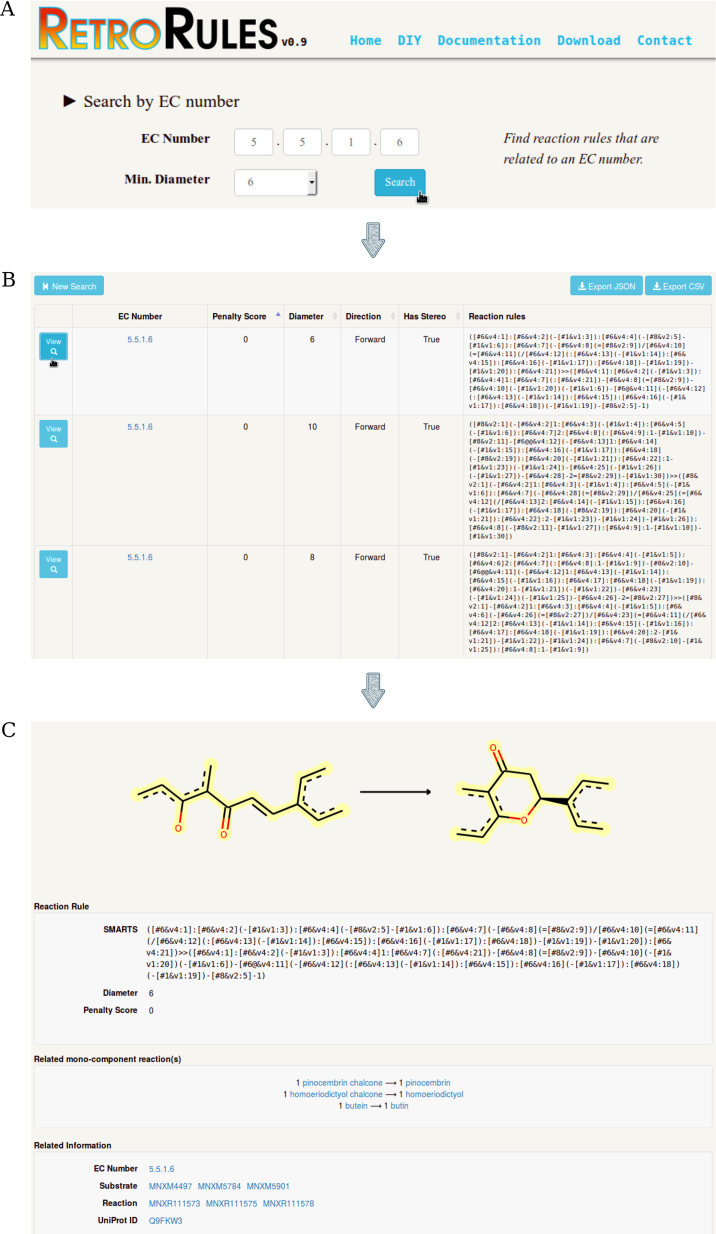
Reaction rules querying process from the RetroRules web interface using as entry point EC number 5.5.1.6 (chalcone–flavanone isomerase) and minimum diameter *d* = 6. (**A**) Home page is the go-to page to start a search for specific reactions and associated rules using EC number, reaction ID, substrate name of structure query. (**B**) Reaction rules matching queries are ranked according to their score in tables that may be exported as CSV or JSON for downstream programmatic analysis. (**C**) Each rule can be further investigated from contextual information such as associated reactions and enzyme sequences using the single reaction rule view.

Using this procedure, two distinct datasets of reaction rules have been generated: one with stereochemistry encoding and another one where stereochemistry description is not included in the reaction rule.

### Stereo reaction rules calculation

Computation of the stereo rules involved similar steps than for the non-stereo rules described previously. Additionally, at step 3 of the procedure, tetrahedral atoms changing their R/S chirality through the reaction (creation, inversion, deletion of chirality) as well as atoms involved in E/Z switch around double bonds are added to the reacting atom set (13). Tetrahedral and E/Z stereochemistry configurations are encoded into SMARTS syntax following the daylight recommendation (http://www.daylight.com/dayhtml/doc/theory/). Release of a comprehensive reaction rule dataset embedding stereochemistry changes was unseen until now to our knowledge. Hence this feature should be regarded as a cutting-edge implementation that is exploratory and still reliant on state-of-the-art stereochemistry perception by cheminformatics packages such as RDKit when used to predict novel products and extend the biochemical space.

### Reaction rules on-the-fly calculation

The custom rule builder available online (https://retrorules.org/diy) stick to the same procedure that the one outlined for precomputed dataset generation, except in two aspects, which are described hereafter. First, all structures of the input reaction are considered as primary compounds, i.e. no filtering will be attempted to remove cofactors. Importance of each structure involved in a reaction for the resulted rules is therefore up to the user to decide. To help in this, a second difference with the general procedure is that the custom rule builder allows for unbalanced reactions, i.e. reactions where the number of atoms differs between left and right-hand sides.

### Reaction rules scoring

Each reaction rule in RetroRules is associated with a score that estimates its degree of biochemical uncertainty. Intuitively, a rule would have less uncertainty if the rule can uniquely be generated from a single enzyme annotation in the database. A penalty score is defined as log_10_(*n*), where *n* is the number of distinct non-redundant enzyme sequences that generated the given rule. In order to calculate *n*, we followed the hierarchical classifications of reaction rules in RetroRules controlled by both the diameter and EC annotation that progressively describe reactions in finer detail (see (4) for details). Such type of penalty score finds application both in order to assess the specificity of a given rule with respect to their annotated sequences and for ranking pathways that were predicted and enumerated through retrosynthesis algorithms.

### Reaction rules on-the-fly scoring

Penalty scores like the ones described in the previous section are also computed for rules generated from user's custom reactions. First, if the calculated rule already exists within the precomputed dataset, the newly generated rule inherits the precomputed score. In other situations where the newly generated rule does not coincide with any precomputed rule then we used a conservative approach by assigning to that rule the worst score value of the database at the considered diameter. The rationale is that our knowledge about that reaction rule is at least as bad as the worst case in the database.

## CONCLUSIONS

RetroRules is a database of reaction rules for metabolic pathway discovery and metabolic engineering. Reaction rules are generic descriptions of reactions to be used in retrosynthesis workflows in order to enumerate possible biosynthetic routes connecting target molecules to precursors. The use of such rules is becoming increasingly important in the context of synthetic biology applied to *de novo* pathway discovery as well as in systems biology to discover underground metabolism and predict new metabolic functions arising from enzyme promiscuity (18). RetroRules provides a complete set of stereochemistry-aware reaction rules spanning >16 000 biochemical transformations, extracted from public databases, and expressed in the community-standard SMARTS format, augmented by a rule representation at different levels of specificity. Such multiple representation of reactions expands natural chemical diversity by predicting *de novo* reactions of promiscuous enzymes. Rules can be directly plugged into bioengineering design tools such as RetroPath2.0 (4) and Selenzyme (5), as well as cheminformatics tools such as the RDKit library http://www.rdkit.org/. Reaction rules can be queried based on product, allowing finding rules that can produce some target compound. Moreover, reaction rules are scored based on enzyme sequence availability, allowing prospective assessment and ranking of pathways.

Metabolic engineering, synthetic biology, and biotechnologists in general will benefit from state-of-the-art tools for pathway and biosensor discovery and design; researchers in biology, biochemistry, and life science in general will gain access to a tool providing insights into enzyme promiscuity and associated biological processes and mechanisms such as those of toxicity and disease. In summary, RetroRules is a far-reaching reaction rules database expected to have a positive impact on the large target user community.

## FUTURE DEVELOPMENTS

The RetroRules database is a public downloadable resource facilitating the task of extracting custom reaction rules subsets for different purposes. In that way, specialized subsets of RetroRules can be anticipated to find application in several fields such as metabolic engineering (central metabolism, plant secondary metabolism etc.), extended metabolic space (underground metabolism, enzyme promiscuity), drug metabolism, xenobiotics, pollutants degradation, bioremediation, sensing enabling metabolic pathways (as in SensiPath (19)), among others. Similarly, the rules can be used to extend metabolic maps of organisms (as in XTMS (20)) or to propose new putative metabolites to annotate mass spectrometry spectra using tools such as OpenMS (as in (21)). Future developments will include expanding the scope of our reaction rules with the addition of the BRENDA database (22) or other community-driven biochemical knowledge resources.

## Supplementary Material

Supplementary Data
